# Supplementary material for: Are carbon emissions trading and green financial instruments synergistic? -Comprehensive quantitative research based on content analysis
Source: PLoS One. 2024 Mar 7;19(3):e0298601. doi: 10.1371/journal.pone.0298601 (PMC10919601; doi:10.1371/journal.pone.0298601)
Supplement: S1 Data — (ZIP) [file pone.0298601.s001.zip › Data/地级市Z_F工作报告.zip!/╔┬╬≈/╤╙░▓╩╨/└┤╫╘╣1⁄2╓┌║┼í╛┬φ┐╦╩2╛▌═°í┐_2014.docx]

各位代表：

现在，我代表市人民政府向大会报告工作，请予审议，并请市政协委员和其他列席人员提出意见。

一、2013年工作回顾

过去的一年，我们紧紧围绕市四届人大三次会议确定的目标任务，坚持以统筹城乡发展总揽工作全局，积极应对经济下行压力和特大自然灾害影响，全力以赴稳增长、调结构、惠民生、抓重建，经济社会保持平稳发展。完成生产总值1354.1亿元，增长6.5%，人均超过1万美元；财政总收入469.7亿元，增长5.7%；地方财政收入155.4亿元,增长11.6%；全社会固定资产投资1321亿元，增长28%；城镇居民人均可支配收入27643元，农民人均纯收入8681元，分别增长11.7%和13.4%；单位生产总值能耗下降3.5%。

经济保持平稳增长。生产原油1691万吨，增长0.3%，加工原油1018.9万吨，下降4%；天然气产量11亿立方米，增长15.8%。生产原煤4114.3万吨，增长20%；卷烟37万箱，净增2.2万箱。非油工业增长18.1%，高于规上工业12.7个百分点。粮食实现“十连丰”，总产74.4万吨；苹果总产244万吨；蔬菜总产106万吨；新增规模养殖户601户，猪牛羊饲养量分别达到150.2万头、19.2万头、96.3万只；红枣、花椒、核桃等产业持续增长。服务业增加值增长10.1%。来延游客和旅游综合收入分别增长30%和28.7%；全社会消费品零售总额增长13%。

结构调整初显成效。延炼永炼汽柴油升级、志丹天然气净化厂、安塞风电等一批重大项目建成。志丹、黄陵、安塞等工业集中区加快建设，全市新增入园企业50户，累计达到309户。净增“四上”企业97户、小微企业3200户，非公有制经济增加值占GDP比重达到19.7%，比上年提高1.2个百分点，增幅居全省第二。启动了60个现代农业示范园建设，建成20个。建成国家级洛川苹果批发市场一期工程。新增省级农业龙头企业8家。十大革命旧址保护提升工程和五大文化旅游园区建设加快推进，建成西北局纪念馆。游客满意度排名第38位，比上年提高18个位次。文化产业增加值增长28.8%。第三产业增加值占GDP比重达到19.8%，比上年提高1个百分点。

城镇化步伐明显加快。中心城区改造全面展开，搬迁山体居民3.3万人。延河大桥加宽、马家湾南滨路等一批市政项目建成投用。完成新区北区一期岩土工程，造地1.8万亩，市级行政中心、新老城连接道路、场地绿化、安置房等项目启动实施。城市建设和用地管理机制不断健全，“双违”整治成果进一步巩固。深入开展全国文明城市创建活动，城市文明程度指数测评在全国61个地级提名资格市中提升到第33位。县城、重点镇和新型农村社区完成投资198亿元，承载能力和公共服务水平明显提升。子长、安塞、黄陵被评为全国卫生县城。5.29万农民进城落户,城镇化率达到54.03%。

民生保障水平稳步提升。在财政增收困难和刚性支出不断增加的情况下，坚持将财政支出和新增财力的80%以上用于民生。全市财政民生支出238.4亿元，增长16.5%。就业工作进一步加强，新增城镇就业1.71万人，农村劳动力转移就业30万人，城镇登记失业率3.64%。社会保障体系更加完善，实现城乡居民医疗保险全市范围异地即时结算；在全省率先实施城乡居民大病保险制度，6575人报销大病保险7200万元；城乡60周岁以上失独家庭扶助标准由每人每月140元分别提高到1000元和800元；实施第三轮母亲健康工程，33.9万人受益。开展居家养老社区服务试点，建成各类养老场所96个，新增床位1030张。八一敬老院新建项目建成入住。向12.98万70周岁以上老人发放高龄补贴1.1亿元。义务教育均衡发展全面推进，整合城区教育资源，推行小学校际联盟和学区化管理，入学难和大班额问题初步缓解。完成学前教育三年行动计划，建设幼儿园66所。医疗保障能力继续提高，县级公立医院改革不断深化，门诊、住院人次增加15%以上，住院次均费用下降20%左右。组建延安医疗集团，启动延安大学附属医院对市第二人民医院和洛川、志丹、延长三家县医院托管。开工91个基层医疗卫生机构基础设施建设项目。扶贫开发力度不断加大，35个扶贫移民搬迁安置点全部启动建设，建成房屋7117套，搬迁入住876户、3434人；扎实推进千名领导干部包扶低收入村活动，筹措资金13.9亿元，实施包扶项目4926个；建立了扶贫专项基金；9.26万人实现脱贫。保障房建设走在全省前列，新开工保障房22831套，建成28749套，新增租赁补贴2000户。文化惠民工程有效实施，建成4个县级文体活动中心和一批乡镇文化站，为430多个村和社区配送了文化器材。举办了陕北民歌大赛等群众文化活动。

基础设施和生态环境持续改善。延志吴高速、东过境公路建成通车。新机场、延延高速、黄延高速扩能工程等项目加快推进。南沟门水库、黄河引水工程进展顺利。建成一批110千伏输变电工程。启动新一轮退耕还林工程，完成造林116.6万亩。治沟造地10.2万亩。新建沼气池5000口，农村沼气入户率居全省首位。解决了13万农村人口安全饮水问题。启动王窑水库水体安全和库区环境综合治理。开展PM2.5监测，城区空气质量优良天数达到327天。超额完成减排任务。甘泉、黄陵被评为全国绿化模范县，志丹被评为国家水土保持生态文明县。

改革开放深入推进。投融资体制改革取得新进展，累计有27家金融保险机构在我市设立分支机构，成立小额贷款公司35家，全年融资31.6亿元。与中建、中铁建等大企业合作实施18个BT项目，总投资超过40亿元。启动行政审批流程再造，精简下放60项审批事项。全面完成撤乡并镇工作。积极推进城乡建设用地指标增减挂钩试点，争取周转指标6309亩。收储土地7112亩，出让商业用地1212.8亩，收缴出让金19.7亿元。招商引资取得新进展，签订合同项目248个，到位资金411亿元，增长33.5%。进出口总额9597.5万美元，增长44.9%，增幅居全省第一。

社会大局保持稳定。建立信访维稳风险评估、领导干部接访双向预约等工作机制，妥善处理了一批信访突出问题。中省交办案件报结率100%，实现“一控双降”目标。坚持抓好道路交通、消防、危化品、森林防火等安全监管，安全生产事故死亡人数和经济损失双下降。加强社会治安防控体系建设，持续开展严打整治专项行动，群众安全感和满意度进一步提升。深入开展“六五”普法，全民法治观念进一步增强。驻延部队和国防后备力量建设不断加强，双拥共建活动深入开展。

抢险救灾和灾后重建取得扎实成效。去年7月份我市遭受百年不遇持续强降雨特大自然灾害，我们把保障群众生命安全放在第一位，紧急转移撤离群众78.7万人，妥善安置受灾群众23.9万人，最大程度降低了灾害损失。大灾之后迅速展开灾后重建，7.4万住房倒损户有4.1万户实现长久性安置，3.3万户实现过渡性妥善安置，保证了受灾群众安全温暖越冬。基础设施、工农业生产和群众生产生活秩序在较短时间内恢复正常。在抢险救灾和灾后重建中，各级党委政府沉着应对、科学指挥，广大基层干部身先士卒、冲锋陷阵，各级各部门尽职尽责、通力协作，社会各界情系灾区、鼎力相助，全市人民群众以坚定的信念、坚强的意志和坚韧的毅力，取得了抢险救灾的重大胜利和灾后重建的阶段性成果，受到国务院领导、省委省政府以及全市人民的充分肯定和高度评价。

政府自身建设不断加强。自觉接受人大、政协和社会各界监督，向市人大常委会报告了贯彻执行《预算法》等方面工作，与市政协专题协商了退耕还林工作，及时向老干部、各民主党派、各界人士通报经济社会发展情况。办理人大代表建议130件、政协委员提案393件。建立市政府领导联系基层群众、人大代表和政协委员制度，组织开展了12次公民代表走进市政府活动。认真执行中央八项规定，全市行政事业单位公用经费压缩7%，“三公”经费下降17.5%，会议费下降37%。建立了事业单位逢进必考制度和临时聘用人员管理制度，党政机关单位人力资源管理不断规范。审计监督力度加大，政府资产实现统一监管，财政投资项目超概问题得到有效控制。开通了政务服务投诉咨询热线，建成社区便民服务中心77个，便民服务点995个。

各位代表，过去一年的成绩，是在经济形势错综复杂、发展面临严重困难的情况下取得的，这是党中央、国务院和省委、省政府关怀支持的结果，是全市人民在市委的坚强领导下，团结拼搏的结果，是市人大、市政协监督支持的结果。在此，我代表市人民政府，向全市干部群众、驻延部队指战员、武警官兵和公安民警致以崇高敬意！向大力支持政府工作的各位代表、政协委员和社会各界人士表示衷心感谢！

在总结成绩的同时，我们也清醒地认识到，我市发展仍面临一些突出矛盾和问题。主要是：产业结构不合理，服务业、非公有制经济发展滞后；县域经济发展不平衡，城镇化质量不高；资源环境刚性约束日益突出，节能减排压力加大；社会治理创新工作有待加强，公共服务能力需要不断提高；“四风”问题不同程度存在，政府行政效能、工作作风和服务水平还需进一步改进。对这些问题，我们将高度重视，下大力气解决，努力把各项工作做得更好，不辜负全市人民的信任和期望。

二、2014年工作的基本思路和预期目标

今年是我市“三年调整、两年提速”承上启下的关键一年，也是加快城乡统筹发展的重要一年。我们将全面贯彻落实习近平总书记系列讲话和十八大、十八届三中全会、省委十二届四次全会、市委四届五次全会精神，坚持以统筹城乡发展为总揽，以结构调整为着力点，全面深化改革开放，加快城镇体系建设，保障改善民生，创新社会治理，加强生态环境建设，扩大投资规模，提升发展速度，推动经济社会健康持续发展。

主要预期目标是：生产总值增长7.5%以上，全社会固定资产投资增长28%以上，地方财政收入增长13%，城镇居民人均可支配收入和农民人均纯收入分别增长12%和14%，城镇化率达到57%以上，单位生产总值能耗下降3.32%。

上述目标是根据当前经济形势和我市发展实际确定的。今年，世界经济形势仍错综复杂，存在许多不确定不稳定因素。我国经济正处于增长速度换挡期、结构调整阵痛期和前期刺激政策消化期叠加阶段，经济下行压力仍然较大。从我市看，以能源为主的经济结构受国内外经济环境和市场变化影响较大，石油产量增幅明显收窄，新的接续产业尚未形成，保持经济持续较快增长面临很大压力。虽然今年预期经济增长速度较上年只提高1个百分点，但要完成这一目标仍需付出艰辛努力。

当前，我市发展也有许多机遇和有利条件。党的十八届三中全会和中央近期召开的一系列会议，对全面深化改革、推动结构调整、加快新型城镇化和农业现代化作出了全面部署，国家继续推进西部大开发战略，推动丝绸之路经济带建设，实施陕甘宁革命老区振兴规划，为我们加快经济结构调整、推动城乡统筹发展带来了新的机遇。全省经济增长仍处于上升通道，将对各地发展形成有力带动。我市近几年全面推进统筹城乡发展，结构调整初见成效，城镇化步伐明显加快，人民生活水平稳步提高，生态环境持续改善，基础保障能力不断提升，加快发展的势能和潜力逐步释放。今年将成为我市经济增长的转折年、速度提升的起步年、结构调整的突破年，是新一轮较快增长期的新起点。只要我们坚定信心和决心，抢抓机遇，奋力赶超，以时不我待、只争朝夕的精神推动发展，以“功成不必在我”的态度打基础、利长远，以“埋头苦干、低调务实不张扬”的作风扎实工作，就一定能够实现经济增长止降回升目标。

三、2014年主要工作任务

（一）抓住支撑增长的重点，促进经济平稳发展

保持油气煤产量稳定增长。支持延长集团、长庆油田等企业实施油气勘探和产能项目建设，实现石油产量恢复性增长和天然气产量突破性增长，全年原油产量达到1730万吨、加工量1130万吨，天然气产量40亿立方米。建成华龙贯屯煤矿、富县党家河煤矿，加快车村一号煤矿和富县芦村一号、二号煤矿建设，推进子长高台、宜川宜南等煤矿建设前期工作。积极开拓煤炭市场，支持帮助煤炭企业扩大生产销售，煤炭产量达到4500万吨。

促进非油煤工业快速增长。制定政策措施，帮助企业解决建设用地、贷款融资、市场销售等难题。积极对接卷烟增产计划，优化产品结构，力争卷烟产量达到43万箱。落实电力、装备制造、石油机械、制药等企业生产计划，跟进协调服务，鼓励扩能上产，促进在建项目投产达效。发电量达到20亿千瓦时，生产载货汽车2600辆。

推动现代农业加快发展。全面落实强农惠农富农政策，持续增加农业投入。以现代农业示范园区为载体，带动“33155”农产品生产基地建设。粮食面积稳定在300万亩，新增苹果面积10万亩、设施蔬菜2万亩。新启动建设14个现代农业示范园区。大力扶持发展龙头企业、农民专业合作社、家庭农场等新型经营主体，新建一批规模化、标准化种养基地和养殖小区。坚持打好洛川苹果、延川红枣、延安小米、延安地椒羊四大特色品牌，不断提高农产品影响力和市场占有率。加快现代农业装备和农技服务体系建设，推进农业信息入村。加强气象灾害监测预警，提高农业防灾减灾能力。

实现现代服务业突破发展。制定推动现代服务业发展的政策措施，启动安塞沿河湾物流产业园区、李渠物流园区、洛川商贸园区建设，建成一批综合批发市场、土特产品专卖店、农家店和33个重点镇超市。加快培育“四上”企业，推动更多经营户“个转企”。积极推进商贸餐饮连锁企业属地注册和大型综合市场统一结算。启动电子商务服务和监管平台建设，推动电子商务加快发展。以4G网络运营为契机，促进信息消费升级。实施城乡家庭服务一体化建设项目，支持家政服务业发展。积极组织各类消费促进活动，落实带薪休假制度，大力培育文化、休闲、健身、养老、金融等新兴服务业。保持市场物价稳定，物价指数涨幅控制在4%以内。全社会消费品零售总额增长13%，服务业占GDP比重达到21%。

（二）大力推进结构调整，全面加快转型发展

加快工业集中区建设。加大“三区九园”建设力度，推进水电路、仓储物流、生活服务基地等配套设施建设。以园区为载体，继续推进能源转化、精细化工、装备制造、高载能、新能源和农产品深加工等产业发展，加快产业转型升级步伐。支持经开区申报国家级开发区和省级高新技术产业开发区。加快重大工业项目建设，建成黄陵煤矸石电厂、甲醇驰放气制合成氨、吴起风电、黄龙太阳能光伏等项目，开工建设大唐热电联产、子长精细化工园区等项目，继续抓好延长油田伴生气资源综合利用、延安煤油气资源综合利用和经开区产业孵化基地、斯伦贝谢长和基地、深圳轻工产业园等项目建设。

全力推动天然气开发利用。支持延长集团、长庆油田等企业加大天然气勘探开发力度，实现突破性发展。组建延安能源集团，推进气化延安和天然气液化、发电、化工等转化利用项目，启动志丹、宝塔陶瓷工业园区建设。开工志丹、宜川、吴起、子长、宝塔、安塞、延川等县区液化天然气项目建设，液化天然气生产能力达到270万吨，产量达到70万吨。

放手发展非公有制经济。发挥产业结构调整专项基金作用，扶持民营企业发展化工合成材料、化学建材、有机化工、精细化工、塑料制品等能源化工下游产业和石油机械、工程车辆等配套产业。引导民营企业投资发展风能、生物能发电、太阳能光伏、新材料、节能环保等新兴产业。鼓励组建一批民营投资公司，继续推出BT项目，吸引民间资金和社会资本参与建设。支持民营企业整合资源、重组合并，组建石油伴生气开发利用、石油工程服务、装备制造、商业零售等实业集团。支持企业技术创新，新建5家市级工程技术研究中心、3家重点实验室。完善市县中小企业服务平台，帮助破解资金、用地、人才等制约难题，力争非公有制经济占GDP比重达到21%。

培育壮大文化旅游产业。推进十大革命旧址改造提升，完成宝塔山、枣园、杨家岭、凤凰山、西北局等旧址基础设施建设和内部提升，积极推进5A级景区创建。加强与陕文投集团、陕旅集团、延长集团等企业合作，推进黄帝文化园、黄河文化园、黄土风情文化园、桥儿沟文化创意产业园、圣地河谷文化旅游园区建设。加快乾坤湾景区和东方红广场建设，启动中国革命艺术家博物院、中国石油博物馆、延安历史博物馆、延安革命英烈馆等项目建设。坚持开展旅游环境综合整治，完善游客服务中心、酒店餐饮等配套设施，不断提高游客满意度。加大旅游宣传营销力度，促进旅游综合收入稳步增长。

（三）狠抓重大项目建设，保持固定资产投资快速增长

加快项目推进。在抓好产业项目建设的同时，全力推进重大基础设施项目建设。加快新机场、延延高速、黄延高速扩能工程等项目建设，开工建设蒙西至华中铁路延安段、沿黄公路、志丹至南梁公路、黄陵动车站等项目。全面开工建设黄河引水工程，南沟门水库实现汛前蓄水。建成一批输变电项目，继续推进县城、重点镇、新型农村社区气化工程。

加强项目谋划储备。围绕园区建设、产业发展和基础设施改善，加强项目策划和储备。鼓励县区、部门和企业与高校、科研院所、专业机构合作谋划项目，建立多层次、分行业项目储备机制，保证重大项目接替有序。抓住国家规划建设丝绸之路经济带的机遇，做好项目准备工作，争取纳入中省规划。积极争取中省继续支持我市灾后重建项目。

继续扩大招商引资。突出陶瓷产业、精细化工、生产性服务基地和央企非能源项目四个重点领域，制定招商方案，出台激励政策，创新招商机制，努力实现招商引资新突破。完善招商项目落地协调推进机制，夯实县区、部门招商项目推进责任，优化项目建设环境和服务保障，提高招商项目履约率和开工率。招商引资到位资金达到500亿元，其中引进省外资金300亿元，分别增长25%以上。

（四）以人的城镇化为核心，大力推进城镇体系建设

加快中心城市建设改造。围绕建设陕甘宁革命老区中心城市目标，大力推进中心城市建设改造。全面加快新区建设，抓好市级行政中心、市民服务中心、住宅小区、学校医院、道路等项目建设，启动二期岩土工程。继续加快旧城改造，启动七里铺、南门坡、大东门、向阳沟等片区改造和安置小区建设，推进清凉山、宝塔山、桥儿沟等区域居民下山。加快市区绿化和水电路气热等市政项目建设，规划建设一批具有地域文化特色的城市雕塑和景观小品，展示古城风貌，彰显圣地特色，让城市延续历史、融入自然，努力建设生态宜居城市。

加强城市精细化管理。扎实开展全国文明城市创建活动，以治脏、治乱、治污为重点，进一步优化城市环境。积极推进全国“智慧城市”试点，完善数字城管信息系统，全面推行网格化管理，建立反应快速、处置及时、运转高效的长效管理机制。继续开展市区广告牌匾专项治理。强化城市规划和土地管理，落实四级监管责任，严肃查处违法建设、违法用地行为。加强窗口行业管理和市民文明道德教育，提升旅游城市形象。

提升城镇承载服务功能。坚持走新型城镇化道路，推进县城、重点镇、新型农村社区协调发展，促进产业与城镇融合发展，提高城镇容纳人口、吸纳就业和综合承载能力。加快实施县城扩容、居民下山、城中村改造和绿化美化等项目，打造宜居环境，提升城市品位。完善重点镇、新型农村社区基础设施、公共服务和产业园区配套，加快人口聚集，实现重点镇、新型农村社区基本定型。

推进农业转移人口市民化。健全进城农民就业、养老、教育、医疗、社保、住房等保障机制，使进城农民平等享受市民待遇。实行农民工与城镇居民同等的住房保障制度，启动“城市建设者之家”公寓建设。将农民工子女纳入义务教育经费保障和普惠性学前教育、免费中等职业教育范围。全面放开县城、建制镇户籍限制，有序放开市区落户限制，做好进城农民落户工作，提高户籍人口城镇化水平。

（五）按照保基本可持续原则，进一步提升民生保障水平

努力做好就业工作。以高校毕业生、城镇生活困难人员、农村转移劳动力等为重点，千方百计扩大就业。创新小额担保贷款工作，以创业促就业。推进就业服务信息化、动态化管理。整合培训资源，加强劳务技能培训。健全完善劳动关系协调机制，强化劳动保障执法和调解仲裁，构建和谐劳动关系。制定民营企业吸纳就业的鼓励政策，多渠道解决就业难题。城镇新增就业1.6万人以上，农村劳动力转移就业28万人以上，大学毕业生初次就业率80%以上。

进一步完善社会保障体系。完善城乡养老、医疗保险制度一体化，继续扩大人群覆盖面。建立城乡居民基础养老金正常调整机制。完善大病保险制度，实现基本医保、大病保险和大病救助无缝对接。实现生育保险市级统筹，推进工伤保险扩面提标。推行居家养老社区服务模式,探索建立社区家庭医生服务机制。健全关爱农村“三留守”人员服务体系。认真落实“单独二孩”生育政策。健全孤儿保障制度，加快儿童福利机构建设。坚持为残疾人办好实事。推进殡葬改革，加快城乡公益性公墓建设。

继续抓好灾后重建。加快城镇和集中安置区住房重建，确保受灾群众入冬前全部搬入新居。完善集中安置点水电路气和垃圾污水处理等基础设施。启动实施避灾移民搬迁五年规划，今年完成4万户、14万人搬迁。加快实施学校、医院、乡镇机构、革命旧址、道路、库坝和工农业生产设施等重建项目，提升经济社会发展和人民生活水平。

着力解决困难群体住房难题。开工建设保障房40500套，棚户区改造22500户，新增发放租赁补贴2000户。积极开展利用住房公积金贷款支持保障房建设试点工作。推进廉租房和公租房并轨运行。加强居民小区物业监管，建立社区介入物业公司评议制度和物业公司淘汰退出机制，提升物业管理服务水平。

加大扶贫开发力度。坚持整体推进与精准到户相结合，加强贫困人口信息化、动态化管理，用好扶贫专项资金，解决无劳动能力贫困人口生活困难，扶持有劳动能力贫困人口发展产业。坚持扶贫移民搬迁向重点镇集中安置，全年搬迁10346户、36388人。坚持开展千名领导干部包扶低收入村活动，广泛动员社会各界参与扶贫开发，努力营造专项扶贫、行业扶贫、社会扶贫“三位一体”工作格局，实现8.05万贫困人口脱贫。

办好人民满意的教育。优化教育资源配置，加强素质教育，促进义务教育均衡发展。深化中小学“四制”改革，全面推行教育视导制度，加强教师培训，提高教育教学质量。启动第二轮学前教育三年行动计划。理顺市区两级办学投入机制，落实各级办学责任。继续推进城区教育资源整合，完善城区小学校际联盟运行机制，推行中学学区化管理和校际联盟，逐步解决择校、大班额问题。与北师大和北京第二实验小学合作，在新区建设中学和小学。鼓励和规范社会力量办学。建成重点镇标准化学校7所、幼儿园4所。实现义务教育营养改善计划全覆盖。实施好特殊教育提升计划。重视职业教育发展，提高城乡劳动力就业创业能力。

切实解决群众看病难问题。巩固县级公立医院改革成果，完善城乡居民医疗保险政策，合理解决群众医疗负担问题。促进延安医疗集团实质性运行，实施县级公立医院托管重点镇卫生院，推动优质医疗资源下沉和城乡医疗卫生服务一体化。探索实施市级公立医院改革。新建市中医院、妇女儿童医院、精神病医院，改扩建市传染病医院，启动建设市口腔医院。加快市县和县乡医院之间远程诊疗信息系统建设，提高卫生信息化水平。继续实施好基层卫生机构基础设施建设项目。加强民营医疗机构监管。开展乡村医生培训，稳定基层医疗队伍，提高服务能力。

保障群众饮食用药安全。大力开展食品安全示范店、示范街和放心县创建活动，抓好肉、奶、豆制品和食盐等重点食品专项整治。完成肉菜追溯体系建设，实现城区肉菜追溯全覆盖。实施放心粮油全覆盖工程，建成市县两级配送中心和100个乡镇、100个农村经销店。启动市区豆制品、熟食品加工园区建设，推进各县区食品加工园区建设。加强学校食堂、校园周边小饭桌管理，强化各类医疗机构和零售药店监管。将食品药品质量安全纳入县乡政府绩效考核，严格落实监管责任，让群众吃得安全放心。

提升城乡供水保障能力。加快实施王窑水库水体安全和库区环境综合治理工程，完成王窑水库供水管线北过境路改线、红庄水库渗漏治理和管线改造、西川河应急供水工程建设，加快市自来水厂扩能改造，新增日供水能力2万方。积极推行中水回用、雨水积蓄，启动延河水利用项目，逐步推行城市分类用水和阶梯水价。完成6个县城和9个重点镇供水工程建设，解决16万农村人口安全饮水问题。

努力缓解城市交通拥堵。加快城市道路内外环线建设，打通一批断头路，促进市政道路、市区主干线、高速公路畅通连接。建设改造一批交通设施，建成龙湾和杨家岭隧道，完善城市交通标志标识。扩建一批停车设施，缓解市区停车难问题。启动公交总公司和汽车东站迁移，规范公交车、出租车、客运车管理，增加运营车辆，延伸运营线路，延长运营时间，方便群众出行。

丰富群众精神文化生活。实施文化强市战略，加强文艺精品生产，保护非物质文化遗产，推进陕北文化生态保护实验区和市级非遗生产性保护示范基地建设，深度开发红色文化、历史文化和民俗文化资源。继续推行“三馆”免费开放，完成市电影院综合楼主体建设，启动市广播电视台新建项目。加强社区文化场所和体育基础设施建设，坚持开展春节文化、文艺调演、全民健身等活动，丰富群众精神文化生活。

（六）全面深化改革，进一步增强发展活力

严格按要求推进中央顶层设计的重大改革，扎实推进中省已明确部署的改革，突出问题导向，结合我市实际，积极稳妥推进中省鼓励先行先试的改革。

启动国家统筹城乡综合配套试验区改革。争取国家批复我市设立统筹城乡综合配套改革试验区，在革命老区开发建设、城乡一体化、资源型经济转型、区域合作开发及财税体制、融资体制、企业信用监管体系等领域先行先试，积极承担中省改革试点任务，探索启动一些深层次改革课题,解决统筹城乡发展中的突出问题。

大力推进行政审批制度改革。完成审批流程再造，全面推行并联审批、网上审批、限时办结、重大项目全程代办、集中办证等制度，提高办事效率。加大简政放权力度，继续取消下放一批行政审批事项和审批权限。推进工商登记制度改革，创造更加宽松的企业准入环境。完成食品药品监督管理体制改革和卫生计生机构合并。积极推进教育、卫生行业事业机构分类改革。

全面推进财税体制改革。加快实施“营改增”试点工作。大力推行财政专项资金管理改革，全面实行部门事业费、专项经费项目化管理。理顺市县财政分配关系，健全城镇基本公共服务支出分担机制，优化市对县转移支付结构，建立社区运转经费财政预算制度，增强基层政府公共服务保障能力。建立新区管委会、城改办土地经营收益和建设投入封闭运行机制。创新行政事业单位国有资产经营管理，组建国有资产交易中心，实现国有资产经营权和使用权统一交易。

积极推进人事管理制度改革。坚持事业单位逢进必考制度，全面推行机关事业单位临时聘用人员劳务派遣制度。严格控制机构编制，财政供养人员只减不增。加强市属国有企业人事管理。

继续深化投融资体制改革。依托各类融资平台，进一步推动重大项目融资工作。鼓励民间资本组建投融资集团，继续支持发展小额贷款公司、村镇银行等金融机构，加快组建延安农村商业银行。推动发行二期城投债券和短期融资券，鼓励具备条件的县区发行城市建设债券。加快启动助保贷业务，解决中小企业发展资金短缺问题。

深化农村综合改革。扩大农村土地经营权确权试点，推进土地承包经营权流转，鼓励支持治沟造地、农民承包土地、林地、撂荒地向专业大户、家庭农场、农民合作社和龙头企业流转，发展适度规模经营。探索建立农村土地经营权、宅基地使用权、集体林权等产权抵押融资机制，推动农村资源和农民资产资本化、市场化。

推进用地管理改革。大力促进城乡建设用地增减挂钩周转指标市域流转和农村集体经营性建设用地流转入市。建立城乡统一的土地资源公共交易平台，实行国有经营性土地公开拍卖，提高土地资本经营收益。

加大国有企业改革力度。力争完成市直国企改制任务。鼓励民营企业通过多种方式参与国有企业兼并、重组，发展混合所有制经济。建立职工托管中心，解决好职工安置、生活保障和就业创业问题。探索建立国有企业资产经营公司，促进国有资产保值增值。

（七）围绕生态延安目标，加强生态环境建设

坚持不懈抓好生态建设。深入开展国家森林城市创建活动，扎实推进新一轮退耕还林，坚持封山禁牧，巩固生态建设成果。实施好天然林保护、三北防护林等林业重点工程，加快市区、县城以及重点镇、新型农村社区、铁路公路沿线和村庄、黄河峡谷绿化，完成绿化造林90万亩。加强农田水利建设和水土流失治理，高标准高质量推进治沟造地，新造地10万亩。

强化大气和水污染防治。继续推进市区锅炉煤改气，推广居民燃气分户壁挂炉取暖。加强汽车尾气排放治理，实行城市规划区机动车环保绿标管理，鼓励各类汽车实施油改气。以建设工地、渣土运输、裸露土地为重点，集中开展扬尘污染治理。全面启动延河综合治理。加快市污水处理厂扩容改造和县区污水处理厂提标改造，坚决制止在河道乱排乱放，实行县区出境水质断面考核制度。加强油区环境综合治理和油气管线安全管理，严防石油污染和安全事故发生。

加强节能减排。继续推进国家环保模范城市创建和低碳城市试点工作。完善建设项目节能评估制度，加强重点用能单位节能管理，推广油田环保节能和煤炭清洁利用技术。大力发展节水农业，推广节水工艺和器具，建设节水型社会。积极推广太阳能光伏照明、节能建筑材料等产品。完成主要污染物减排任务。

建设美丽乡村。加强农村规划建设管理，启动实施33个重点镇和100个新型农村社区、高速公路、铁路、国省道沿线、城乡结合部环境综合整治，整体推进村落改造，实施垃圾污水处理、道路硬化、河道整治、绿化美化等工程。加强文化旅游名镇、古镇古村落保护，建设一批具有浓郁黄土气息、体现陕北文化特色的村镇。

（八）创新社会治理，维护社会和谐稳定

积极预防和化解社会矛盾。完善信访工作考核和责任追究机制，落实信访三级终结制度，下功夫解决信访突出问题。深入开展社会矛盾纠纷排查，健全完善信访、调解、复议、诉讼协调互动机制，畅通群众诉求渠道，着力从源头上化解矛盾。

坚持抓好安全生产。建立“党政同责、一岗双责、齐抓共管”工作体系，全面落实政府、部门监管责任和企业主体责任。强化重点领域专项整治，及时消除各类隐患。严格落实建设项目安全设施“三同时”制度，坚持推行挂牌督办、警示约谈、黄牌警告、绩效考核和“一票否决”制度。健全防灾减灾和应急管理体系，完善突发事件处置预案，加强应急演练和专业抢险队伍建设，提高各类灾害、事故和突发事件应急处置能力。

强化社会治安管理。深化“三官一律”进社区和社区民警专职化工作，开展群防群治，推行网格化管理，提高社会面管控水平。加强社会稳定风险评估，推进立体化防控体系建设，强化重点区域和公共场所监管，依法严厉打击“两抢一盗”、“黄赌毒”等犯罪活动，不断提高人民群众安全感。重视保护妇女儿童合法权益。加强国家安全和保密工作，依法管理民族宗教事务。大力支持部队建设，推进军民融合式发展，巩固双拥共建成果。

四、加强政府自身建设

坚持依法行政，建设法治政府。自觉接受人大及其常委会的法律监督、工作监督和人民政协的民主监督，认真办理人大代表、政协委员建议提案，坚持向人大常委会报告工作、与政协协商工作制度。健全完善重大决策专家论证、公众参与、风险评估、集体决定机制。充分发挥工会、妇联、共青团等人民团体的桥梁纽带作用，主动接受人民群众和媒体监督。加强执法队伍建设，完善执法人员持证上岗和资格管理制度，促进规范公正文明执法。

扎实开展群众路线教育实践活动，促进作风转变。自觉遵守中央八项规定、国务院约法三章和省委、市委相关要求，坚决整治“四风”，以良好的作风推动工作。加强调查研究，精简会议文件，减少事务性活动。全面推行群众和服务对象监督评价部门及窗口单位制度，强化行政问责和效能监察，大力整治不作为、慢作为、乱作为和庸懒散、生冷硬等问题。健全完善督查考核机制，促进工作落实，提高行政效能。

推进政务公开，增强政府工作透明度。完善政务公开制度，拓展政务公开范围。加强市县政务中心标准化建设，全面推行一个窗口受理、一站式审批、一条龙服务运行机制和群众办事干部代办制度。加强电子政务公共服务平台建设，积极推行网络问政，及时办理网民留言，方便群众知情、参与和监督。继续开展公民代表走进政府和大学生到政府机关见习活动，搞好市民热线办理，让群众监督权力、让权力在阳光下运行。

加强政府廉政建设，树立清正廉洁形象。严格落实党风廉政建设责任制，不断完善政府系统廉政建设长效机制。坚决停止新建、扩建、迁建或购置政府性楼堂馆所，坚持行政经费“零增长”、“三公”经费只减不增。严格执行住房、车辆配备等规定，继续开展公车治理和清理违规占用办公用房活动。坚持用制度管权管事管人，加强财政资金、土地出让、工程建设、招投标等重点领域行政监察和审计监督，坚决查处违法违纪案件，努力建设廉洁政府。

各位代表，延安正处在调整结构、转型发展的关键时期，任务艰巨而繁重，责任重大而光荣。让我们在市委的坚强领导下，团结拼搏，迎难而上，以更加振奋的精神，更加扎实的工作，全力推进统筹城乡发展，为建设圣地延安、生态延安、幸福延安而努力奋斗！
